# Iohexol Clearance for Determination of Glomerular Filtration Rate in Rats Induced to Acute Renal Failure

**DOI:** 10.1371/journal.pone.0123753

**Published:** 2015-04-13

**Authors:** Michelle T. Passos, Sonia K. Nishida, Niels O. S. Câmara, Maria Heloisa Shimizu, Gianna Mastroianni-Kirsztajn

**Affiliations:** 1 Department of Medicine, Division of Nephrology, Federal University of Sao Paulo, Sao Paulo-SP, Brazil; 2 Department of Immunology, Institute of Biomedical Sciences, University of Sao Paulo, Sao Paulo-SP, Brazil; 3 Department of Nephrology, University of Sao Paulo—School of Medicine, Sao Paulo-SP, Brazil; University of Sao Paulo Medical School, BRAZIL

## Abstract

**Introduction:**

The glomerular filtration rate (GFR) is considered an especially important tool for the measurement of renal function. Inulin clearance (InCl) is the classic reference method for this purpose, although it is associated with a number of disadvantages; thus, other markers have been proposed, including iohexol. Determination of iohexol clearance (IoCl) has been established for clinical use; however, its application as a GFR marker in experimental rat models has not been reported.

**Objectives:**

This study aims to standardize a methodology for the measurement of iohexol clearance and to evaluate its applicability as a marker of GFR in rats with induced toxic acute renal failure (ARF), using InCl as the gold standard.

**Materials and Methods:**

Twenty-six Wistar male rats (200–300 g) were divided into the following two groups: a control group (n=7) and an ARF group (n=19). ARF was induced by the subcutaneous administration of cisplatin (5 mg/kg); IoCl and InCl were determined simultaneously, and plasma creatinine (pCreat) dosage was measured colorimetrically.

**Results:**

The pCreat, InCl and IoCl levels were consistent with the expected values for the renal function ranges of the evaluated animals, and the IoCl and InCl levels were significantly correlated (r=0.792). An inverse moderate linear correlation between the IoCl and pCreat measurements (r=-0.587) and between the InCl and pCreat measurements (r=-0.722) were observed.

**Conclusion:**

These results confirm a correlation between IoCl and the gold standard of GFR, InCl measurement. IoCl offers a relevant advantage over InCl because determination of the former allows the animal to live after the procedure.

## Introduction

Glomerular filtration rate (GFR) is considered the best parameter for the evaluation of renal function. Because there are methodological obstacles to determining this rate, markers that can be easily used without the loss of accuracy would be very useful in clinical practice and in experimental studies involving animal models.

Since its introduction in 1935, inulin clearance has been the reference method for GFR determination because inulin satisfies all of the requisites of an ideal marker of renal function [1–4].

Clinical and experimental determinations of inulin clearance present methodological and analytical problems because such testing requires continuous inulin infusion; catheterization for blood collection, inulin administration and the maintenance of physiological conditions; measurement of the precise time period for urine collection; and in experimental models, anesthesia and bladder catheterization. The latter procedure could result in urinary tract infections and ureter lesions. Additionally, the many analytical methods that are generally adopted for inulin quantification are restricted in terms of usefulness and are cumbersome; moreover, they are associated with laboratory errors [5]. Therefore, different markers have been proposed as alternatives to the use of inulin clearance, including endogenous substances, such as creatinine, and exogenous substances, such as iohexol [5,6].

Iohexol is an iodinated, non-ionic radiographic contrast agent with characteristics similar to inulin, particularly with regard to its use in GFR evaluation [1,5].

The measurement of iohexol clearance has been validated in humans, and its determination shows good correlation with the GFR estimation formula [7]. Additionally, iohexol clearance has been successfully standardized for the assessment of renal function in dogs [8–11] compared with Tc^99m^ DTPA clearance [10] and inulin clearance. This marker has been successfully evaluated in different animals [12–17]; however, there are no reports of its use in studies involving rats.

Our objective was to evaluate the use of iohexol in these animals, which would be very useful because it would not require that the animal be sacrificed, as is the case for inulin clearance.

## Objectives

This study aims to standardize a methodology for the measurement of iohexol clearance and to evaluate its applicability as a marker of GFR in Wistar rats with induced toxic acute renal failure (ARF), using inulin clearance as the gold standard.

## Materials and Methods

Twenty-six male Wistar rats weighing 200–350 g were submitted or not to the ARF induction protocol, which was approved by the ethics committee of the Federal University of Sao Paulo (CEP 0028/11).

To induce ARF, the animals were submitted to subcutaneous administration of 5 mg/kg cisplatin (1 mg/mL). One, 2, 3 and 4 days after cisplatin administration, iohexol clearance and inulin clearance were simultaneously performed, and at the end of these procedures, the animals were sacrificed by the administration of excessive anesthetics.

Two groups of Wistar rats were evaluated as follows: a control group (CT, n = 7) and an ARF group (n = 19). The latter group was divided into four subgroups (groups 1, 2, 3 and 4). For the ARF rats, the clearances were performed 1, 2, 3 and 4 days after cisplatin administration, which were designated G1 (n = 5), G2 (n = 5), G3 (n = 5) and G4 (n = 4), respectively.

### Creatinine

Plasma creatinine (pCreat) was measured by Jaffe’s method utilizing a commercial kit. To standardize the methodology, the iohexol and inulin clearances were both measured in the same animal.

### Iohexol clearance

For the determination of iohexol clearance, iohexol (300 mg/mL) was administered via intraperitoneal injection (IP), and its plasma disappearance was measured at certain time intervals. The animals were anesthetized with sodium pentobarbital at 50 mg/kg of body weight (BW); then, an initial blood sample was collected (time zero), and iohexol was administered through the IP route. Additional blood samples were collected (via the jugular vein) 40, 80, 120 and 140 min after IP injection to construct a plasmatic decay curve of iohexol.

After its collection, the plasma was centrifuged and stored at -20°C. The iohexol concentration was then determined by capillary electrophoresis according to the technique described by Shihabi et al. [18], with modifications. Specifically, a 50 μL volume of plasma, control or standard, was diluted in 150 μL of a normal rat plasma pool. After deproteinization, with the addition of 300 μL of acetonitrile containing 6 μL/mL of 3-isobutyl-1-methylxantine as the internal standard, the supernatant was analyzed by capillary electrophoresis at a 25 kV, 254 nm pressure injection for 3 seconds. A standard curve of iohexol ranging from 5 to 150 μg/mL that was prepared using a normal rat plasma pool was included in all measurements.

### Inulin clearance

After the surgical procedure, a loading dose of inulin (100 mg/kg BW diluted in 0.9% NaCl) was administered through the jugular vein. Subsequently, a constant infusion of inulin (10 mg/kg BW in 0.9% NaCl), at an infusion rate of 0.04 mL/min, was started and maintained until the end of the experiment. Three urine samples were collected at 30 min intervals by bladder catheterization, and blood samples were obtained at the beginning and end of each experiment.

The plasma and urine were measured using the anthrone method, with modifications.

The iohexol and inulin clearances are expressed as mL/min/100 g BW.

### Statistical analysis

Descriptive analyses and tests (Student’s t-test, the point [19] and interval [20] estimates of Pearson’s correlation coefficient, and the point and interval estimates of the intraclass correlation coefficient [21]) were utilized when appropriate. The Bland-Altman test was used to measure agreement between the numerical variables of the different clearances that were compared in the present study. The significance level was considered α = .05.

## Results

Iohexol determination exhibited linearity from 0 to 400 μg/mL, and the intra-assay variation coefficient was <8.3%, whereas the inter-assay variation coefficient was <5.9%.

The groups studied were apportioned as follows: 26.9% CT rats; 19.2% G1, G2 and G3; and 15.4% G4. Iohexol clearance, inulin clearance and pCreat were measured in all of the rats.

The seven CT rats presented a mean iohexol clearance of 0.780 mL/min/100 g BW and a mean inulin clearance of 0.892 mL/min/100 g BW. The mean pCreat was 0.429 mg/dL.

The 19 ARF rats presented a mean iohexol clearance of 0.354 mL/min/100 g BW and a mean inulin clearance of 0.593 mL/min/100 g BW. The mean pCreat was 1.253 mg/dL.

The measurements of both clearances in CT and in ARF 1, 2, 3 and 4 days after cisplatin administration are presented in Table 1, which shows iohexol and inulin clearances at different levels of renal function.

**Table 1:** Measurements of iohexol clearance, inulin clearance and creatinine (mg/dL) levels in control rats and in rats 1, 2, 3 and 4 days after cisplatin administration.

**Groups**	**Iohexol clearance (mL/min/100 g BW)**	**Inulin clearance (mL/min/100 g BW)**	**Plasma creatinine (mg/dL)**
**CT**	0.789 ± 0.144	0.892 ± 0.099	0.429 ± 0.170
**G1 (day 1)**	0.662 ± 0.136	0.925 ± 0.123	0.560 ± 0.089
**G2 (day 2)**	0.281 ± 0.086	0.713 ± 0.141	1.000 ± 0.187
**G3 (day 3)**	0.225 ± 0.099	0.381 ± 0.126	1.480 ± 0.602
**G4 (day 4)**	0.220 ± 0.091	0.294 ± 0.045	2.150 ± 0.957

**Fig 1:**
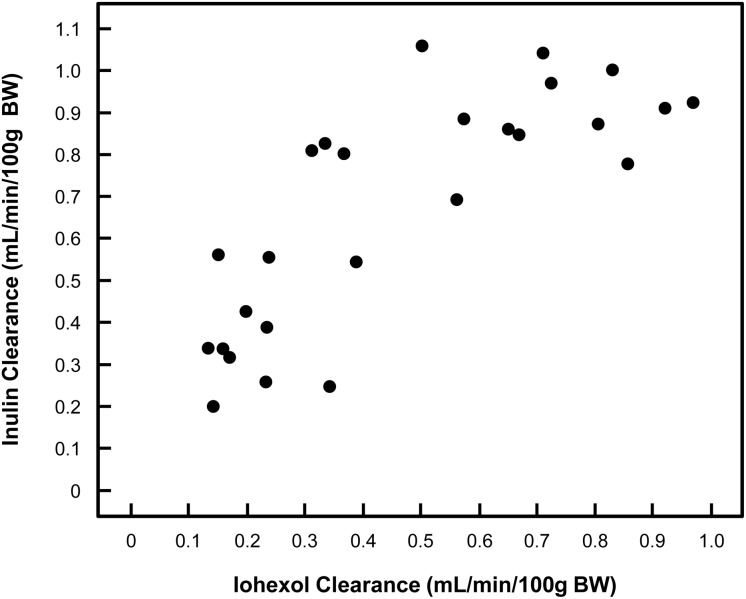
Distribution of inulin and iohexol clearances of all rats (CT and ARF).

As observed in Fig 1, there was a significant correlation between inulin clearance and iohexol clearance (r = 0.792). The CT group had higher iohexol clearance (p<0.001) and higher inulin clearance (p = 0.001) as well as lower pCreat (p<0.001) compared with the ARF group.

Statistically significant correlations were observed between inulin clearance, iohexol clearance and pCreat, as shown in Table 2.

**Table 2:** Estimates of Pearson’s linear correlation coefficients.

**Parameters**	**Coefficient** ^a^	**CI (coefficient)** ^b^	**P**
**pCreat vs. iohexol clearance**	-0.587	[-0.794; -0.258]	0.002
**pCreat vs. inulin clearance**	-0.722	[-0.867; -0.465]	<0.001
**Inulin clearance vs. iohexol clearance**	0.792	[0.584; 0.902]	<0.001

CI, confidence interval.

^a^ Pearson’s linear correlation coefficient.

^b^ Interval of the 95% confidence for Pearson’s linear correlation coefficient

The concordance analysis was concluded with an estimate of the intra-class correlation coefficient. When the CT and ARF results were grouped, the inferential results indicated a statistically significant concordance between inulin clearance and iohexol clearance (p = 0.001).

To adjust the iohexol clearance to better estimate the inulin clearance, we investigated which would be the best statistical model to apply for clearance adjustment, and the quadratic model demonstrated the best adjustment for both CT and ARF. The evaluation was performed according to the sample dimension (Table 3).

**Table 3:** Estimates of the quadratic models that established a relationship between inulin clearance and iohexol clearance for the CT and ARF groups.

	**CT Group (n = 7)**			**ARF Group (n = 19)**		
**R** ^**2**^	0.786			0.651		
**Model**	Coefficient	Standard error	p	Coefficient	Standard error	p
**Constant**	-1.404	0.757	0.137	-0.069	0.168	0.686
**Iohexol**	5.685	2.015	0.048	2.864	0.901	0.006
**Iohexol** ^**2**^	-3.414	1.310	0.060	-2.081	0.967	0.047

## Discussion

Pursuant to the inadequacy of inulin clearance as a practical test for the assessment of GFR, several studies have evaluated iohexol clearance as a useful marker in experimental models, allowing standardization in different species.

Rats are very important animals for experimental studies in the context of kidney diseases, and new and simpler tests for the determination of GFR that are applicable to this group are of great interest.

In this study of the evaluation of iohexol clearance at different levels of renal function involvement, the animals were submitted to a protocol of ARF induction by the administration of cisplatin, a potentially nephrotoxic medication.

The observations of the development of post-cisplatin ARF reinforced the findings of previous studies; increases in the plasma creatinine levels occurred progressively, reaching their maximum on the 4^th^ day after the administration of cisplatin, corresponding to the point at which GFR reached its nadir in the ARF group.

The absolute levels of iohexol and inulin clearance were consistent with those expected for the GFR ranges of the CT and ARF groups, whose linear correlation was moderate in magnitude and statistically significant (r = 0.792).

In addition, the inulin and iohexol clearance values were consistently close to each other along the range of measurements, with lower values of iohexol clearance paralleling those of inulin clearance. Because the difference between the methods was uniform, estimating the GFR (i.e., “estimate” the inulin clearance based on the iohexol clearance results) should be possible when utilizing an adjustment index. Thus, adjustment equations were developed for the CT and ARF groups that allowed more precise determination of the GFR, thereby approximating the obtained iohexol clearance results to those of inulin clearance and leading to the conclusion that the quadratic model was the most adequate model for this purpose. The corresponding equations demonstrated how such an adjustment might be utilized in further studies. Nevertheless, we are not able to definitively confirm the established adjustment factors because of the limited number of animals that were evaluated.

Certain aspects relative to the tests performed with iodinated substances should be accepted with care [10], and among these are the following: the measurement error of the iodine content of the contrast agent, which is typically approximately +2%, and the measurement error of the injected volume, which is typically approximately +1%. Considering these potential sources of error, the details provided when describing the methodological standardization were given special attention, such as by correcting the real volume of iohexol that was administered.

Iohexol can cause deleterious effects on kidney tubules, but certainly the amount of iodinated contrast media administered is an important determinant of such effects in experimental animals, as reported by Caglar et al. [22], who administered intravenous injections of 1, 3 or 9 g iodine per kg body in rats. This group demonstrated, by ultrastructural observations, that the effect of the contrast media was mainly on the proximal convoluted tubule cells, but it also occurred in other parts of the nephron as well. Additionally, as the dose was increased, the biochemical analysis became more altered.

Because of the extra renal clearance of the markers, plasma clearance methods (iohexol, Cr^51^ EDTA, among others) generally overestimate the true GFR, as shown in dogs [6,23]. In such studies, the levels were underestimated when compared with those of inulin clearance. A potential explanation for this finding is the occurrence of the saturable tubular reabsorption of iohexol that has been documented in dogs in an in vitro perfusion study [24]. This species-specific peculiarity might explain the greater difference in the normal value ranges of the GFR (the CT animals) between iohexol vs. inulin. These plasma levels are lower than in animals with renal function deficits, and tubular reabsorption would be more expressive, considering the percentage of the filtrated load.

Our study showed an adequate intra-class correlation in the ARF group but not in the CT group, and the absence of correlation in that group could be explained by the small number of animals included in the group.

The determination of GFR by the measurement of iohexol clearance showed concordance with the gold standard, but the new method has certain advantages. The methodology is simple and involves a semi-automated determination. Overall, it allows the animal to remain alive, creating the possibility that each animal might be its own control, thereby addressing the physiological differences that exist between animals.

At last, in addition to its applicability in experimental studies, as exposed here, it is important to highlight the role of iohexol clearance in special populations such as obese individuals [25], children [26], patients with AIDS [27], and others in whom it is particularly difficult to establish the real glomerular filtration rate; furthermore, an easier technique can contribute to clinical management.
